# A solid-phase approach for the synthesis of α-aminoboronic acid peptides†

**DOI:** 10.1039/c7ra13479g

**Published:** 2018-01-16

**Authors:** Blake E. Daniels, Craig E. Stivala

**Affiliations:** Discovery Chemistry, Genentech, Inc. 1 DNA Way South San Francisco CA 94080 USA stivala.craig@gene.com

## Abstract

A solid-phase synthesis of α-aminoboronic acid peptides using a 1-glycerol polystyrene resin is described. Standard Fmoc solid-phase peptide chemistry is carried out to construct bortezomib and ixazomib. This approach eliminates the need for liquid–liquid extractions, silica gel column chromatography, and HPLC purifications, as products are isolated in high purity after direct cleavage from the resin.

## Abstract 2

A solid-phase synthesis of α-aminoboronic acid peptides using a 1-glycerol polystyrene resin is described.

## Graphical abstract

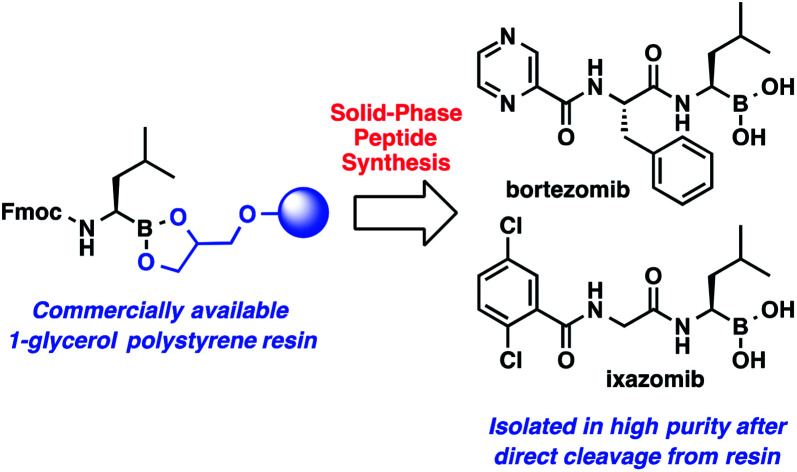

α-Aminoboronic acids are currently being investigated for their utility as reversible covalent inhibitors in a diverse range of therapeutic applications (Fig. 1).^1^ These compounds' Lewis acidity enables the formation of stable tetrahedral adducts with nucleophilic residues in biological targets (Fig. 2). In 2003, the first boronic acid drug, bortezomib, was approved for the treatment of multiple myeloma.^2^ Ixazomib, a related α-aminoboronic acid inhibitor, was later approved in 2015 for the same indication.^3^

**Fig. 1:**
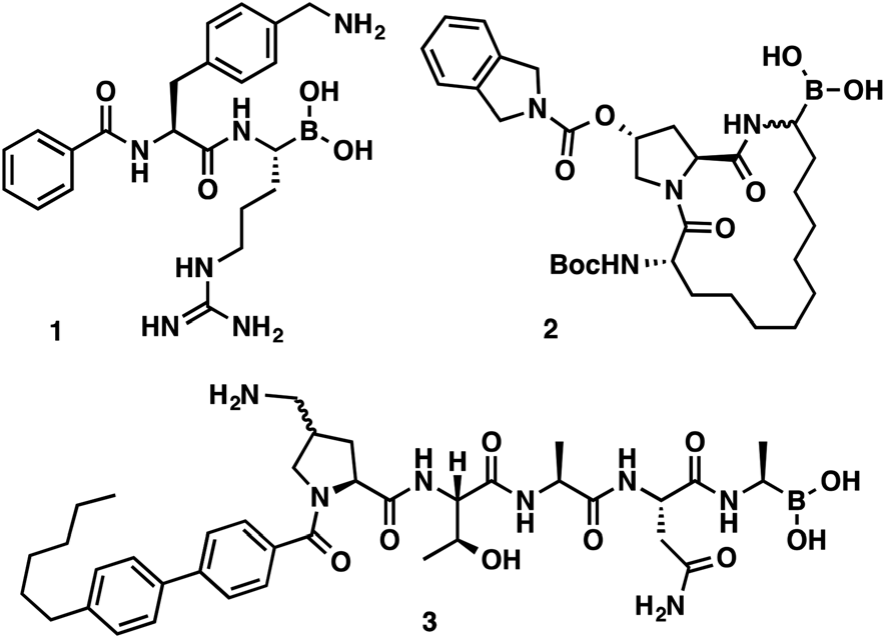
α-Aminoboronic acids featured in various drug discovery programs. Flaviviral protease inhibitor (1), HCV protease inhibitor (2), LepB inhibitor (3).

**Fig. 2:**
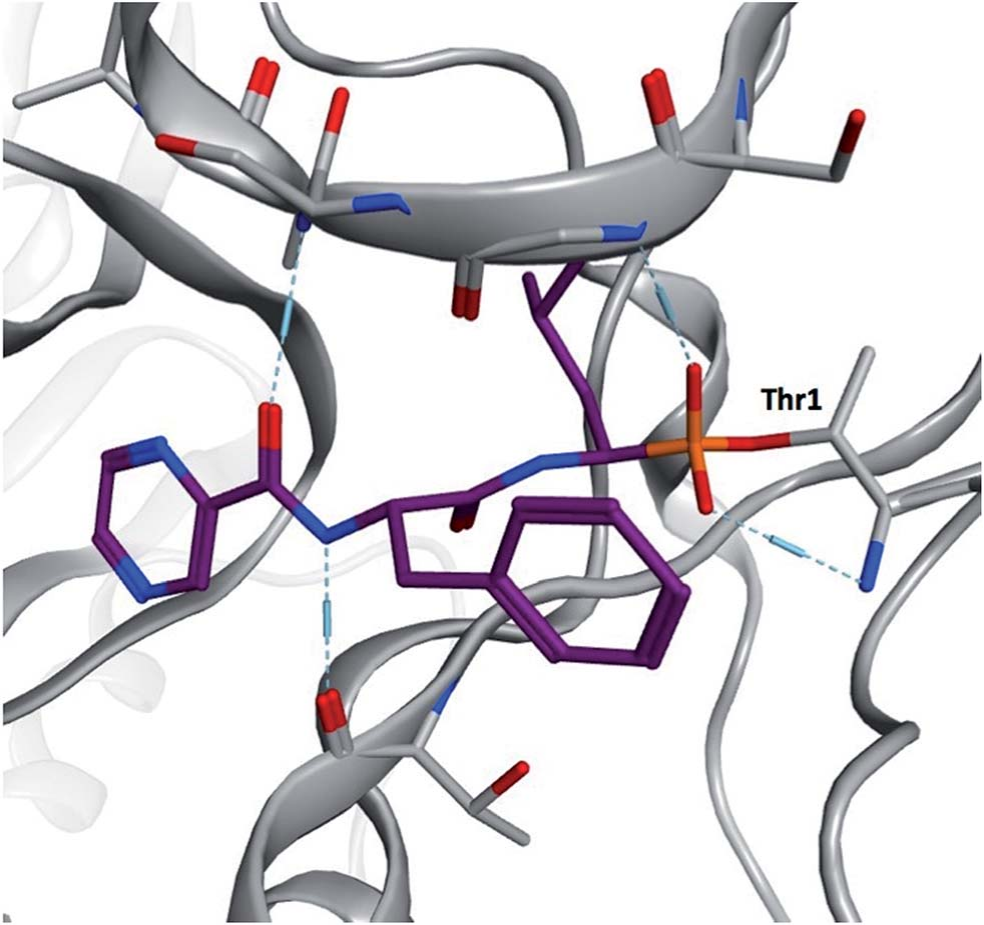
Yeast 20S proteasome in complex with bortezomib (PDB ID 2F16). The boronic acid forms a stable tetrahedral adduct with the N-terminal threonine (Thr1).

Peptidic α-aminoboronic acids, such as bortezomib and ixazomib, have traditionally been assembled using standard peptide coupling techniques,^4^ wherein an α-aminoboronic ester is introduced onto a pre-constructed peptide and is subsequently deprotected to unmask the boronic acid. Metal-catalyzed decarboxylative borylation strategies have also been reported for the preparation of α-aminoboronic acid peptides.^5^ This approach provides direct access to these compounds from their parent peptide constructs but sacrifices stereochemical integrity.

Regardless of the method, α-aminoboronic acid/ester peptides are difficult to prepare for a number of reasons.^6^ First, the C–B bond can be oxidatively labile.^7^ Second, α-aminoboronic acids and esters containing an unsubstituted α-amino group can undergo a spontaneous 1,3-rearrangement (Scheme 1, A); this process can be minimized or suppressed entirely if the amino group is rapidly acylated or protonated.^8^ Third, boronic esters can be hydrolytically labile, especially at low pH (Scheme 1, B).^6,9^ Therefore, any multistep approach must entail careful extractive workups and purifications to ensure that the ester remains intact.

**Scheme 1:**
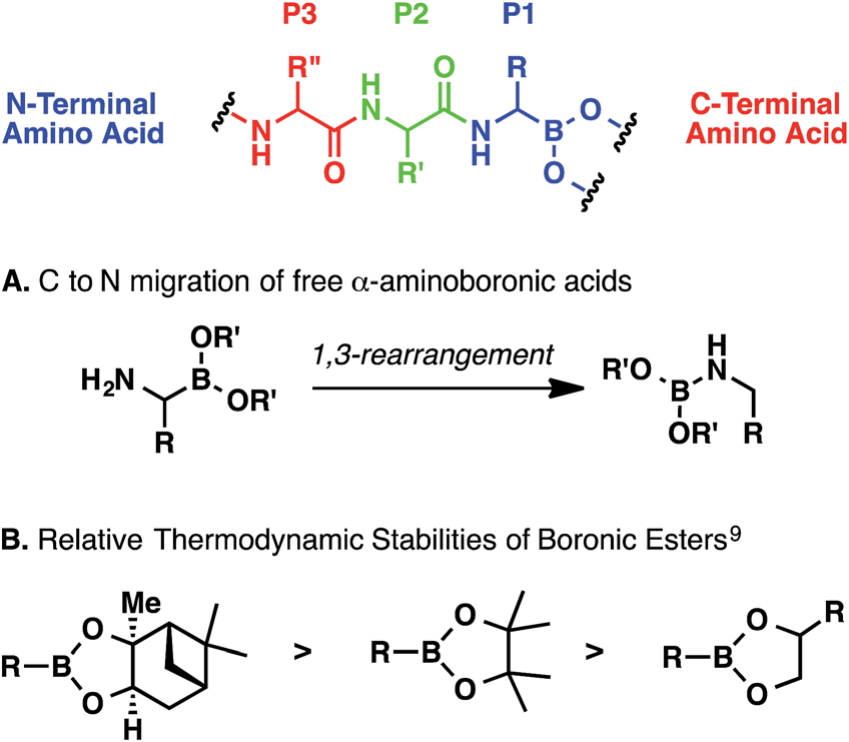
General considerations for preparing α-aminoboronic acid peptides.

While solid-phase peptide synthesis (SPPS) has become a standard method for the construction of peptides,^10^ this technology has remained underexplored for the preparation of α-aminoboronic acid peptides.^11^ An approach of this type could eliminate liquid–liquid extractions and HPLC purifications and could enable high-throughput access to this class of compounds. To the best of our knowledge, there has only been one report of C-terminal SPPS to generate α-aminoboronic acid peptides (Scheme 2).^12^ Although this study provides a critical conceptual foundation, the approach it describes lacks the simplicity of a traditional solid-phase approach, requiring a complex 8-step synthesis to prepare resin-bound α-aminoboronic ester 6 for SPPS. This limitation may preclude its use as a general strategy for the preparation of α-aminoboronic acid peptides.

**Scheme 2:**
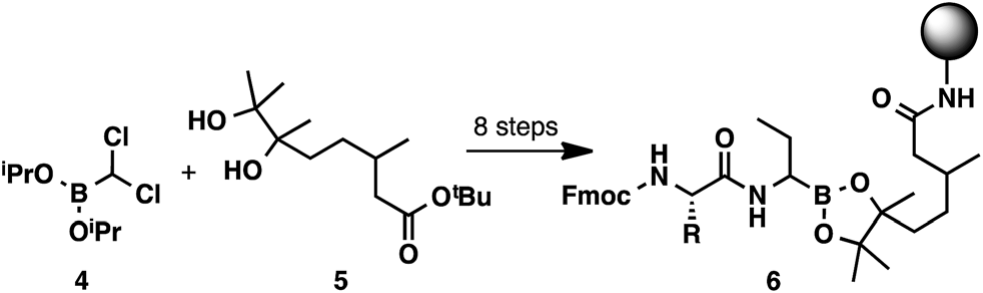
8-Step preparation of a resin-bound α-aminoboronic acid for C-terminal SPPS.

We sought to identify an approach that could enable access to resin-bound α-aminoboronic acids for SPPS in a limited number of steps using the emerging supply of commercially available α-aminoboronic acid building blocks. The Klein group recently described the use of a 1-glycerol polystyrene resin that could be used for Fmoc SPPS to construct boronic acid-containing peptides.^13–15^ These results prompted us to explore the use of this resin for preparation of α-aminoboronic acid peptides, specifically bortezomib and ixazomib.

Considering the unique reactivity of α-aminoboronic acids, we needed to devise a concise loading strategy that would suppress the potential for C to N boron migration. This required the amine to remain protonated or acylated throughout the loading process. These considerations lead to the design of a two-step loading protocol (Scheme 3). Commercially available boroleucine pinanediol ester 7 was hydrolysed with aqueous HCl. The boroleucine salt (8) was isolated in quantitative yield, free of pinanediol impurities, after a simple liquid–liquid extraction. The crude boroleucine salt was then shaken with the 1-glycerol polystyrene resin (loading capacity 0.6 mmol g^−1^),^13^ Fmoc chloride, and *N*,*N*-diisopropylethylamine to provide resin-bound Fmoc-protected boroleucine 9.

**Scheme 3:**
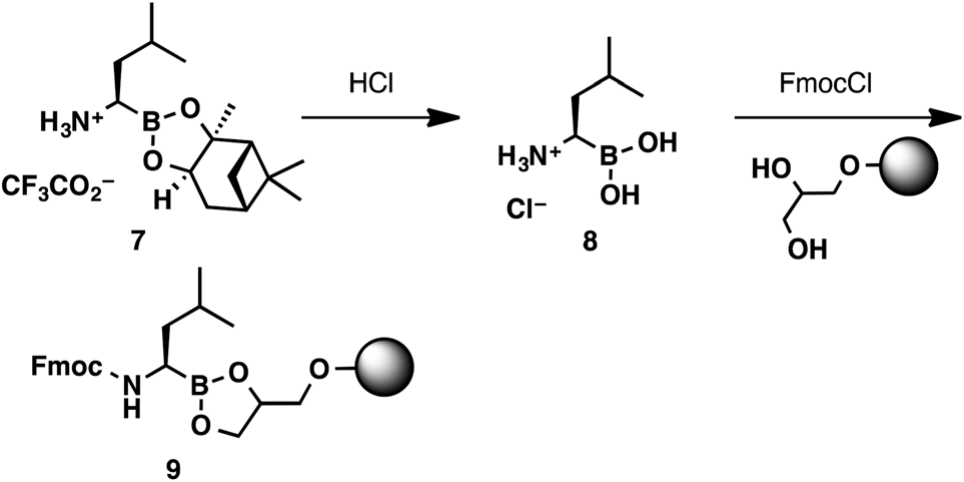
2-Step protocol for loading boroleucine onto a 1-glycerol polystyrene resin.

With the C-terminal α-aminoboronic acid resin in hand, we used standard Fmoc SPPS coupling techniques^16^ to synthesize bortezomib (Scheme 4). Fmoc deprotection (piperidine, DMF) and amide coupling (Fmoc-Phe-OH, TBTU, *N*,*N*-diisopropylethylamine, DMF) delivered intermediate 10. A subsequent Fmoc deprotection/coupling sequence with pyrazinecarboxylic acid produced resin-bound bortezomib 11. Hydrolysis of the resin bound peptide was accomplished with gentle shaking in a THF/water mixture.^13^ Filtration and concentration delivered bortezomib (7-steps from boroleucine pinanediol ester 7) in 54% yield and in >95% purity.

**Scheme 4:**
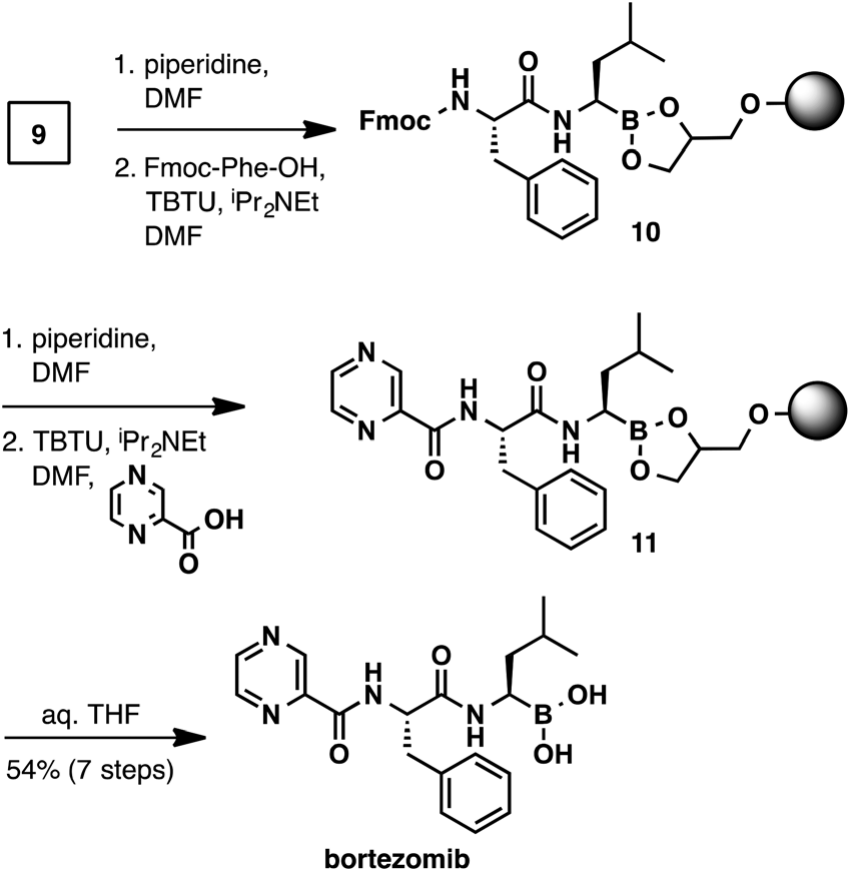
Solid-phase synthesis of bortezomib.

The synthesis of ixazomib (Scheme 5) was accomplished in an analogous manner. Fmoc deprotection (piperidine, DMF) and amide coupling (Fmoc-Gly-OH, TBTU, *N*,*N*-diisopropylethylamine, DMF) delivered intermediate 12. The deprotection/coupling sequence was repeated with 2,5-dichlorobenzoic acid to generate resin-bound ixazomib 13. Finally, boronic ester hydrolysis (THF/water) provided ixazomib in 49% yield and in >95% purity.

**Scheme 5:**
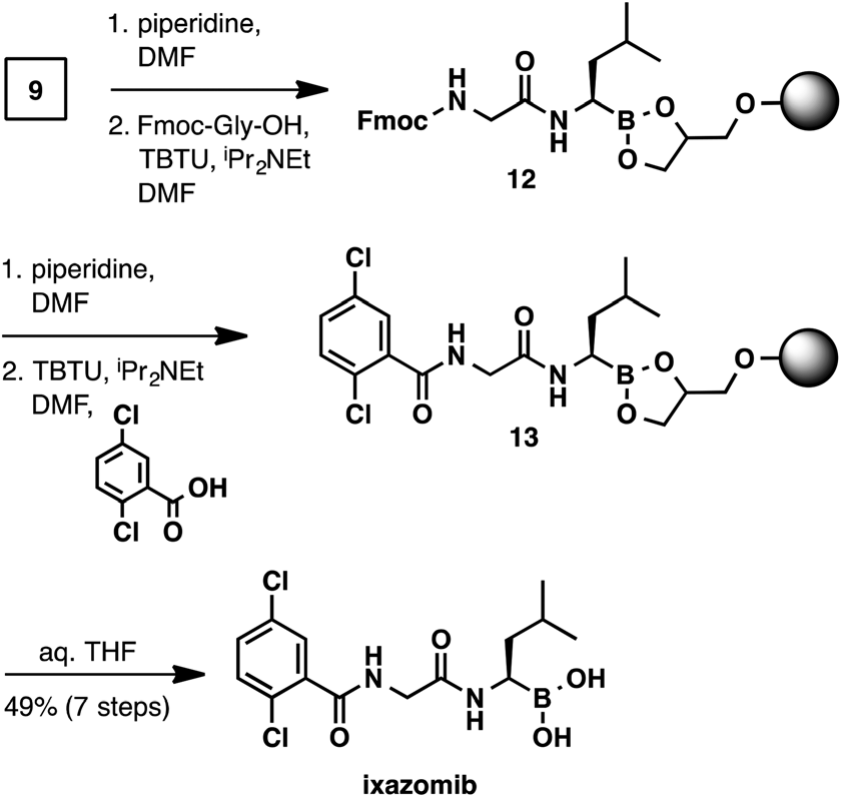
Solid-phase synthesis of ixazomib.

## Conclusions

This proof-of-concept study details the use of the 1-glycerol polystyrene resin for C-terminal SPPS of α-aminoboronic acid peptides. The α-aminoboronic acid used in this study was loaded onto the resin in 2 steps from a commercially available building block. This loading protocol represents a dramatic improvement to what has been reported previously. While the efficiency of the solid-phase synthesis of bortezomib (7 steps from 7, 54% yield) and ixazomib (7 steps from 7, 49% yield) is comparable to other standard synthetic approaches,^4,5^ the use of a solid support enables α-aminoboronic acid peptides to be constructed rapidly in high purity and eliminates the need for workup and purification.

## Experimental procedures

### General

All commercial reagents and anhydrous solvents were used without additional purification. Boroleucine pinanediol ester 7 was purchased from Ark Pharm (Cat. # AK-44948, CAS # 179324-87-9). 1-Glycerol polystyrene resin was purchased from Iris Biotech (Cat. # BR-5206.0025). Nuclear magnetic resonance (NMR) spectra were acquired on a Bruker Avance DPX400 operating at 400 and 100 MHz for ^1^H and ^13^C, respectively, and are referenced internally according to residual solvent signals. NMR data were processed using MNova software and recorded as follows: ^1^H-NMR – chemical shift (*δ*, ppm), multiplicity (s, singlet; d, doublet; t, triplet; q, quartet; m, multiplet), coupling constant (Hz), and integration; ^13^C-NMR – chemical shift (*δ*, ppm). High-resolution mass spectra (HRMS) were recorded on a Thermo Scientific Orbitrap Q Exact mass spectrometer. Reactions were monitored by a Shimadzu LCMS/UV system with LC-30AD solvent pump, 2020 MS, Sil-30AC auto sampler, SPD-M30A UV detector, CTO-20A column oven, using a 2 – 98% acetonitrile/0.1% formic acid (or 0.001% ammonia) gradient over 2.5 minutes. Purity was determined by LCMS analysis with an Agilent 1290 UHPLC coupled with Agilent MSD (6140) mass spectrometer using ESI as ionization source. The LC separation used a Phenomenex XB-C18, 1.7 μm, 50 × 2.1 mm column with a 0.4 mL min^−1^ flow rate. Solvent A was water with 0.1% FA and solvent B was acetonitrile with 0.1% FA. The gradient consisted of 2 – 98% solvent B over 7 min and hold 98% B for 1.5 min following equilibration for 1.5 min. The LC column temperature was 40 °C. UV absorbance was collected at 220 nm and 254 nm.

#### Synthesis of boronic acid 8

(*R*)-Boroleucine-(1*S*,2*S*,3*R*,5*S*)-(+)-2,3-pinanediol ester trifluoroacetate (1.00 g, 2.64 mmol, 1.00 equiv.) and isobutylboronic acid (297 mg, 2.77 mmol, 1.05 equiv.) were suspended in heptane (5.86 mL, 0.45 M). Hydrochloric acid (5.86 mL, 0.45 M) was added, and the reaction mixture was stirred at room temperature for two hours. Upon consumption of the starting material, the reaction mixture was diluted with water and heptane (both 10 mL). The aqueous layer was separated and concentrated under reduced pressure to afford (*R*)-boroleucine hydrochloride 8 (442 mg, quantitative yield) as a white solid. ^1^H NMR (400 MHz, methanol-d_4_) *δ* 2.84 (br s, 1H), 1.78–1.64 (m, 1H), 1.53 (m, 2H), 0.97 (d, *J* = 6.6 Hz, 3H), 0.96 (d, *J* = 6.4 Hz, 3H). HRMS (ESI) calcd for C_5_H_15_O_2_NB [M + H]^+^: 132.1190, found 132.1188.

### General procedure for solid-phase synthesis of α-aminoboronic acids

#### Step 1

Resin loading – 1-glycerol polystyrene resin (loading capacity 0.6 mmol g^−1^, 1.50 g, 20 mmol) was swollen in CH_2_Cl_2_ for 30 minutes and then dried *via* vacuum filtration. Fmoc chloride (556 mg, 2.15 mmol, 3.6 equiv.), (*R*)-boroleucine hydrochloride (100 mg, 0.597 mmol, 1.0 equiv.), THF (10 mL, 0.06 M), and then *N*,*N*-diisopropylethylamine (0.38 mL, 2.15 mmol, 3.6 equiv.) were added to the resin and the reaction mixture was shaken overnight. The resin was dried by vacuum filtration and washed sequentially with THF, CH_2_Cl_2_, and DMF (3 × 10 mL each). Note – Quantitative loading of the α-aminoboronic acid is assumed.

#### Step 2

Fmoc deprotection – piperidine (10% in DMF, 20 mL, 21.8 mmol) was added to the resin and the suspension was shaken for 20 minutes. The resin was dried by vacuum filtration and washed with DMF (2 × 10 mL). This procedure was repeated to ensure complete deprotection.

#### Step 3

Peptide coupling – *N*,*N*-diisopropylethylamine (0.42 mL, 2.39 mmol, 4.0 equiv.), an Fmoc-amino acid (1.79 mmol, 3.0 equiv.), and TBTU (587 mg, 1.79 mmol, 3.0 equiv.) were added to the resin in CH_2_Cl_2_ (10 mL) and DMF (10 mL). The reaction mixture was shaken for two hours. The resin was dried by vacuum filtration and washed with DMF (2 × 10 mL) and CH_2_Cl_2_ (2 × 10 mL).

#### Step 4

Cleavage from resin – the resin was dried by vacuum filtration, washed with DMF, CH_2_Cl_2_, and Et_2_O (3 × 10 mL for each), and dried under vacuum. The dried resin was treated with 9 : 1 THF/water (40 mL) and stirred overnight at room temperature. The resin was filtered and the filtrate concentrated under vacuum to afford the pure α-aminoboronic acid.

#### Bortezomib

Bortezomib – recovered as a white solid in 54% yield (124 mg). ^1^H NMR (400 MHz, methanol-d_4_) *δ* 9.13–9.06 (m, 1H), 8.77–8.68 (m, 1H), 8.65–8.58 (m, 1H), 7.36–7.14 (m, 5H), 4.84–4.67 (m, 1H), 3.23–3.14 (m, 1H), 3.12–3.01 (m, 1H), 2.97–2.87 (m, 1H), 1.47–1.28 (m, 3H), 1.28–1.17 (m, 1H), 0.82–0.73 (m, 6H). HRMS (ESI) calcd for C_19_H_24_O_3_N_4_B [M − OH]^+^: 367.1936, found 367.1925.

#### Ixazomib

Ixazomib – recovered as a white solid in 49% yield (106 mg). ^1^H NMR (400 MHz, methanol-d_4_) *δ* 7.60 (dd, *J*_1_ = *J*_2_ = 1.5 Hz, 1H), 7.49–7.46 (m, 2H), 4.23 (s, 2H), 2.83–2.75 (m, 1H), 1.74–1.61 (m, 1H), 1.42–1.32 (m, 2H), 0.97–0.90 (m, 6H). HRMS (ESI) calcd for C_14_H_18_O_3_N_2_BCl_2_ [M − OH]^+^: 343.0782, found 343.0770.

## Conflicts of interest

There are no conflicts to declare.

## Supplementary Material

RA-008-C7RA13479G-s001

## References

[cit1] Teicher B. A., Tomaszewski J. E. (2015). Biochem. Pharmacol..

[cit2] Paramore A., Frantz S. (2003). Nat. Rev. Drug Discovery.

[cit3] Al-Salama Z. T., Garnock-Jones K. P., Scott L. J. (2017). Target Oncol..

[cit4] Beenen M. A., An C., Ellman J. A. (2008). J. Am. Chem. Soc..

[cit5] Li C., Wang J., Barton L. M., Yu S., Tian M., Peters D. S., Kumar M., Yu A. W., Johnson K. A., Chatterjee A. K., Yan M., Baran P. S. (2017). Science.

[cit6] Hall D. G. , Boronic Acids, Wiley-VCH Verlag GmbH & Co. KGaA, 2006, p. 1

[cit7] Matteson D. S. (1999). J. Organomet. Chem..

[cit8] Laplante C., Hall D. G. (2001). Org. Lett..

[cit9] Roy C. D., Brown H. C. (2007). J. Organomet. Chem..

[cit10] Guillier F., Orain D., Bradley M. (2000). Chem. Rev..

[cit11] Elgendy S., Patel G., Green D., Goodwin C. A., Scully M. F., Husman W., Skordalakes E., Kakkar V. V., Deadman J. J. (1997). Tetrahedron Lett..

[cit12] Dunsdon R. M., Greening J. R., Jones P. S., Jordan S., Wilson F. X. (2000). Bioorg. Med. Chem. Lett..

[cit13] Behnam M. A. M., Sundermann T. R., Klein C. D. (2016). Org. Lett..

[cit14] Merrifield R. B. (1963). J. Am. Chem. Soc..

[cit15] Hall D. G., Tailor J., Gravel M. (1999). Angew. Chem., Int. Ed..

[cit16] Zinieris N., Leondiadis L., Ferderigos N. (2005). J. Comb. Chem..

