# Supplementary material for: A solid-phase approach for the synthesis of α-aminoboronic acid peptides
Source: RSC Adv. 2018 Jan 16;8(6):3343–7. doi: 10.1039/c7ra13479g (PMC9077542; doi:10.1039/c7ra13479g)
Supplement: RA-008-C7RA13479G-s001 [file RA-008-C7RA13479G-s001.pdf]

Electronic Supplementary Material (ESI) for RSC Advances.
This journal is © The Royal Society of Chemistry 2018

Copies of NMR spectra

^{1}H NMR of compound **8**

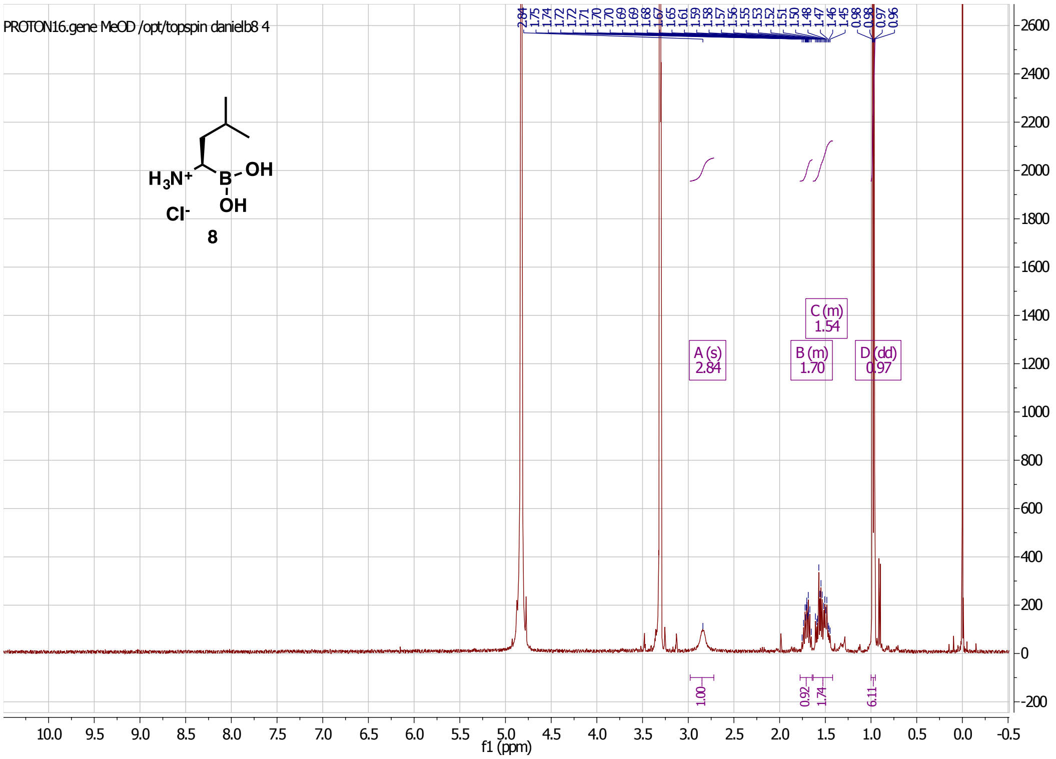

^{1}H NMR of **Bortezomib**

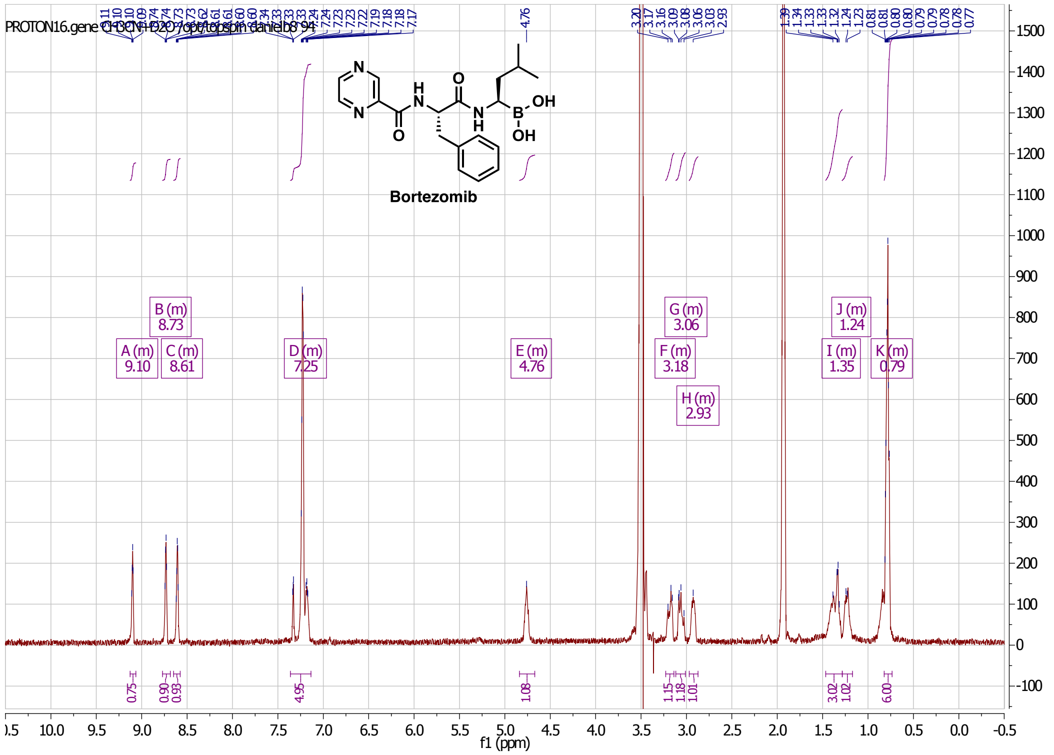

HPLC trace of **Bortezomib**

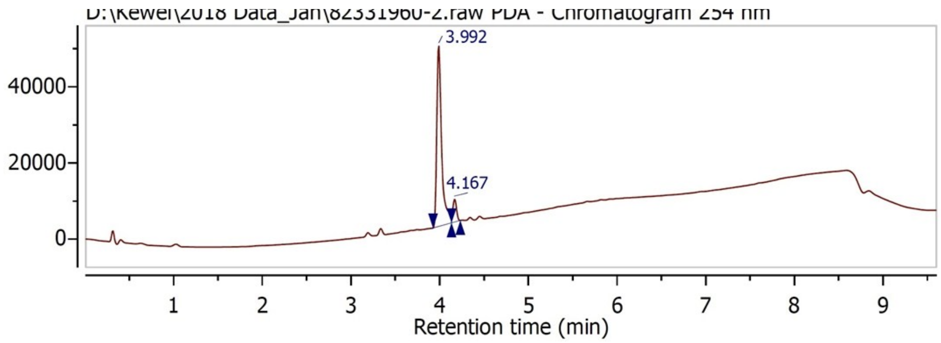

UV 254 (>= 1%)

RT	Area %	MS+
3.99	91.05	367.19
4.17	8.95	367.19

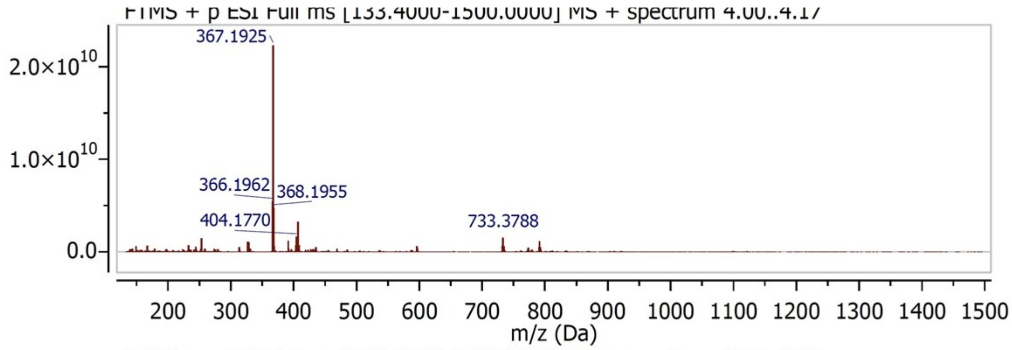

MS of peak at R.T. 3.99

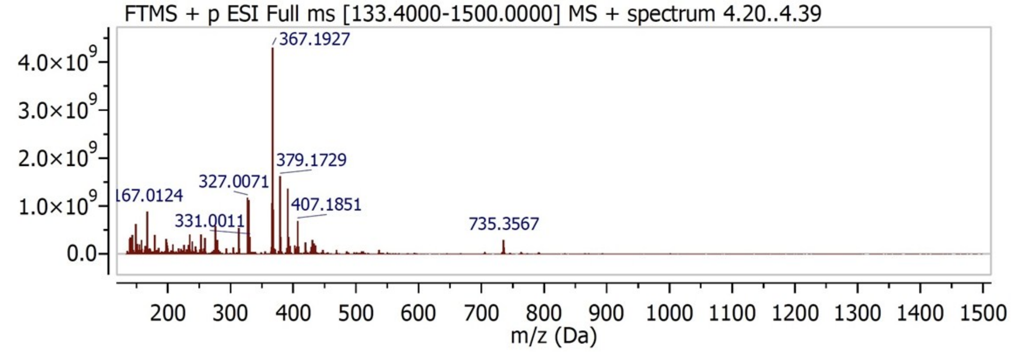

MS of peak at R.T. 4.17

NOTE: Compounds that elute at 3.99 min and 4.17 min are the same. The difference in retention times can be attributed to dimerization or trimerization of the parent boronic acid.

^{1}H NMR of **Ixazomib**

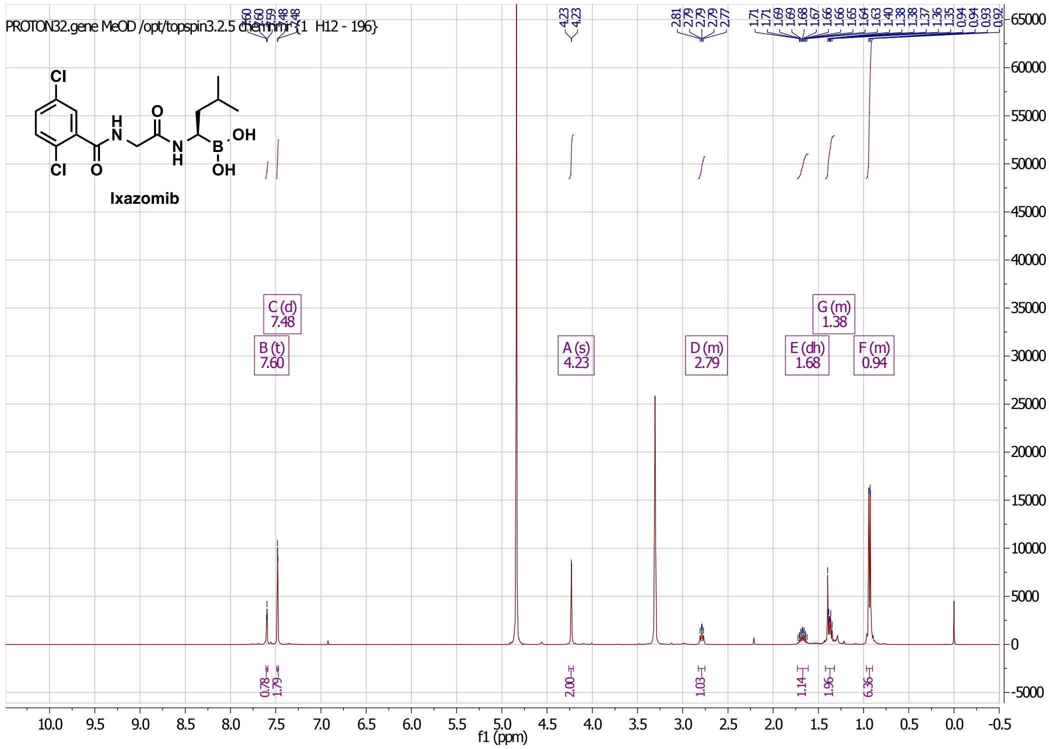

HPLC trace of **Ixazomib**

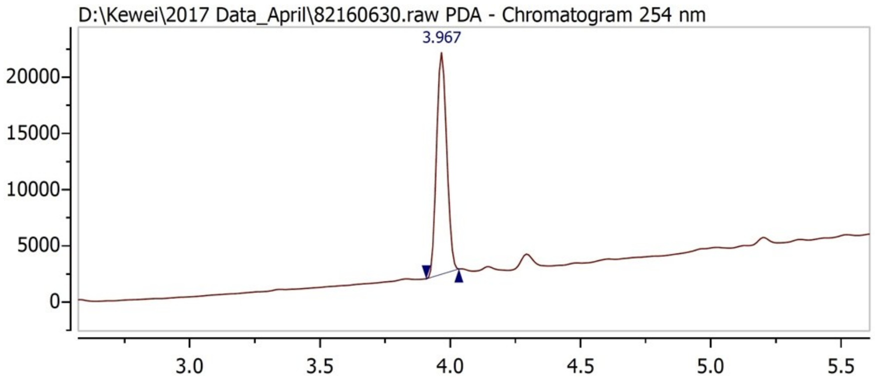

UV 254 (>= 1%)

RT	Area %	MS+
3.97	100.00	343.08

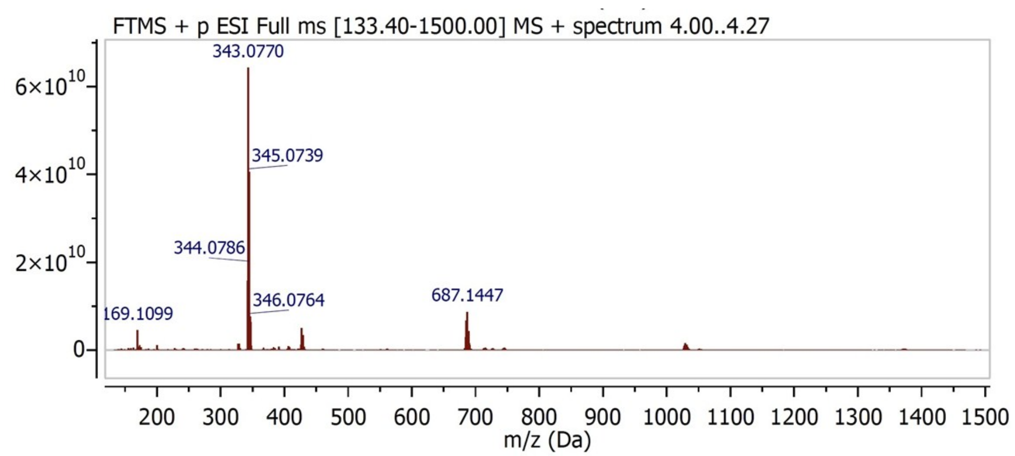

References

1. For ^{1}H NMR of **Bortezomib** see: M. A. Beenen, C. An and J. A. Ellman, *J. Am. Chem. Soc.*, 2008, **130**, 6910–6911.
2. For ^{1}H NMR of **Ixazomib** see: C. Li, J. Wang, L. M. Barton, S. Yu, M. Tian, D. S. Peters, M. Kumar, A. W. Yu, K. A. Johnson, A. K. Chatterjee, M. Yan and P. S. Baran, *Science*, 2017, **356**(6342), eaam7355.
